# Effects of Calendula Essential Oil-Based Cream on Biochemical Parameters of Skin of Albino Rats against Ultraviolet B Radiation

**DOI:** 10.3797/scipharm.1112-18

**Published:** 2012-04-16

**Authors:** Arun K. Mishra, Amrita Mishra, Anurag Verma, Pronobesh Chattopadhyay

**Affiliations:** 1 Central Facility of Instrumentation, School of Pharmaceutical Sciences, IFTM University, Lodipur-Rajput, Moradabad-244001, India.; 2 Institute of Pharmaceutical Sciences and Research Centre, Bhagwant University, Ajmer-305004, India.; 3 Defence Research Laboratory, DRDO, Tezpur, Assam, India.

**Keywords:** Lipid peroxidation, UV-B radiation, Biochemical parameters, Calendula essential oil

## Abstract

Reactive oxygen species (ROS) generated from UV-B radiation have the capacity to cause oxidative decomposition which leads to the formation of toxic components as well as lipid peroxidation. Considering this fact, the present study was performed to evaluate the effect of a cream (O/W) containing the essential oil of Calendula officinalis on biochemical parameters of the skin of albino rats against UV-B radiation. The fingerprint analysis of Calendula essential oil was performed by HPLC with special reference to 1,8-cineole and α-pinene. The results indicated that the treatment with creams containing 4% and 5% of Calendula essential oil caused a significant decrease in the malonyldialdehyde level, whereas the levels of catalase, glutathione, superoxide dismutase, ascorbic acid, and the total protein level were significantly increased after 1 month of daily irradiation and treatment when compared to untreated control groups. The results suggest that the cutaneous application of the essential oil of Calendula prevents UV-B-induced alterations in the level of antioxidants in skin tissue.

## Introduction

Ultraviolet radiation is defined as that electromagnetic radiation with wavelengths between 100 and 400 nm and is divided into UV-A (320–400 nm), UV-B (290–320 nm) and UV-C (100–290 nm). UV-C rays are efficiently blocked by the atmosphere and don’t reach the skin surface. The UV-A rays may penetrate deep into the skin tissue and cause endothelial cellular necrosis, blood vessel damage and collagen degradation. UV-B radiation (having energy that is 30 to 40 times greater than that of UV-A) may promote a deficit in the immunologic functions of the skin in addition to the anomalies in the DNA, thus the abnormal cells that should be destroyed may be tolerated and can divide and replicate giving rise to the formation of cancer [1]. UV-B rays may also cause protein damage, lipid peroxidation and skin lesions [2]. Lipid peroxidation is the process in which free radicals receive electrons from the lipids in cell membranes, resulting in cell damage. Reactive oxygen species (ROS) degrade unsaturated lipids and form malondialdehyde (MDA) which is considered a marker enzyme of lipid peroxidation. The levels of superoxide dismutase (SOD), reduced glutathione (GSH), catalases (CAT), ascorbic acid level (ASC) and total protein level (TP) allow the estimation of the level of antioxidants in the skin tissue

Topical application of substances having antioxidant potential may be able to modulate ROS related signaling pathways induced by UV-B exposure, which might provide protection by interfering with biochemical signals pathways which are causative agents of undesired effects [3]. Therefore, to protect skin against long-term consequences of ROS-generated photodamage, novel materials for protection of skin from UV-B radiation are needed. *Calendula officinalis* is reported to possess a remarkable antioxidant activity, anti-inflammatory activity and wound healing activity [4].

The cosmetic and therapeutic applicability of Calendula is well established especially when concerned with skin-related disorders [5]. The essential oil of Calendula has great potential to inhibit the free radical reactions. A previous study demonstrated that the essential oil of Calendula consists mainly of α-thujene, α-pinene, 1,8-Cineole, dihydrotagetone and T-muurolol [6]. Other constituents of the flowers are sesquiterpenes (e.g. caryophyllene) and triterpenes (e.g. a- and b-amyrins, lupeol and lupenone). Most of the flavonoids present in the plant are the glycoside derivatives of quercetin and isorhamnetion [7, 8]. Mechanism of the possible chemo-preventing action of flavonoids has not yet been completely understood. The soothing effect of Calendula oil is used to cure stomach ulcers and inflammation in form of topical formulations and the flavonoid, triterpene and saponin content may be accountable for this action [9]. The dried petals of the Calendula plant are used in form of tinctures to accelerate the healing of burns, bruises and cuts [10]. Calendula essential oil, if applied externally on the ear, has been reported to alleviate pain and discomfort from an earache [11]. The Calendula essential oil is pale or orange in color with a sweet and strong characteristic aroma. Clinical testing of cosmetic formulations containing the Calendula extract elicited little irritation or sensitization. In our earlier study, we observed almost no skin irritancy in Calendula essential oil based cream formulations [12]. When comparing the Calendula extract and oil, the use of Calendula essential oil to formulate topical formulations imparts many benefits apart from specific pharmacological activity which include pleasant aroma, emolliency and improvement in the elasticity of the skin.

Research studies on phytochemicals, naturally occurring antioxidants, minerals and vitamins have increased at an astonishing rate over the past few years. Thus, considering the fact that Calendula oil may present antioxidant activity, the development of topical formulations containing calendula oil as well as the correct evaluation of biochemical parameters of skin of albino rats against UV-B radiation were investigated. Recent *in vitro* studies from our laboratory have revealed the antioxidant activity of Calendula essential oil based topical formulation [13]. Studies performed on volatile components of basil (*Ocimum basilicum* L.) and thyme leaves (*Thymus vulgaris* L.) exhibited that 1,8-cineole have stronger antioxidant activity than the other components tested in the assay. In the study conducted by Perry NSL et al, the two monoterpenoids (α-pinene and 1,8-cineole) showed good antioxidant activity (inhibition of bovine brain liposome peroxidation) [14]. In a study conducted on the essential oil of *Ricinus communis* (L), compositional analysis revealed the presence of α-pinene, 1,8-cineole, α-thujone, camphor, camphene and finally their antioxidant activity was proven for the essential oil. From this, it was assumed that α-pinene and 1,8-cineole as an antioxidant component of Calendula essential oil may account for balanced biochemical parameters of skin tissue of rats against UV-B radiation.

In the present study, Calendula essential oil was subjected to fingerprint analysis by HPLC and the content of α-pinene and 1,8-Cineole were evaluated. The proposed analytical method was also used to access the recovery of extracted component. Recovery test was performed to evaluate any interaction between component of formulation and Calendula essential oil.

Furthermore, we evaluated the capability of Calendula essential oil based cream (O/W) to protect the skin against UV-B induced biological effects when topically applied *in vivo*. The effects were measured in terms of lipid peroxidation marker enzyme level (MDA) and nonenzymatic antioxidant parameters which include reduced glutathione (GSH), ascorbic acid level (ASC), total protein level (TP) and antioxidant enzymes as superoxide dismutase (SOD) and catalases (CAT). Calendula essential oil is known for its unique composition of flavonoids and terpenes and their pharmacological potential on skin-related disorders. Calendula essential oil has proven its sun protection efficiency, but its effect on biochemical parameters is not well understood. The outcome of the study will assist in elucidating possible mechanisms of action for the effects of Calendula essential oil based cream formulations on biochemical parameters of skin of albino rats against UV-B radiation.

## Results and Discussion

**Fig. 1:**
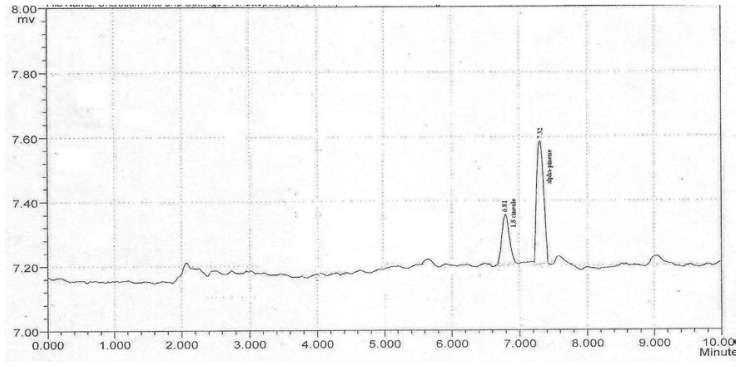
Chromatogram of 1,8-cineole and α-pinene as standard

**Fig. 2:**
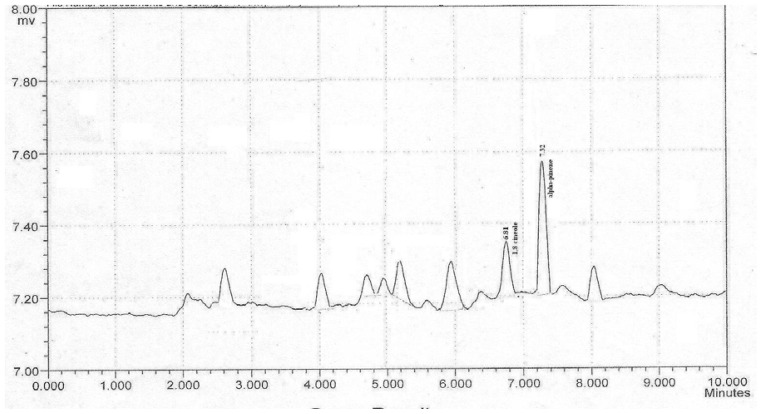
Chromatogram of calendula oil with peaks of 1,8-cineole and α-pinene

The compounds 1,8-cineole and α-pinene are reported as main components of Calendula essential oil and as having proven potential to scavenge free radicals, hence they are selected for the present study. The analysis of two main components of the Calendula oil by HPLC method demonstrated that peaks of 1,8-cineole and α-pinene were clearly resolved and peak symmetry was observed. The retention time (RT) for 1,8-cineole and α-pinene were 6.81 and 7.32min respectively (Fig. 1 and Fig. 2). By employing HPLC method, the concentrations of 1,8-cineole and α-pinene in the essential oil were found to be 8.12±0.7 and 22.53±0.2%, respectively (Tab 1). The analytical method was validated on parameters as linearity, LOD, LOQ, accuracy and precision (interday and intraday) (Tab. 2).

The Calendula essential oil was used to prepare F4 and F5 formulations and then subjected to *in-vivo* study. To assess any possibility of interaction of formulation contents with Calendula oil components, recovery study was performed in triplicate by proposed HPLC method for the two formulations. The recovery of 1,8-cineole and α-pinene in both the formulations was above 95% which indicates no interaction (Table 3). Experimental doses of UV-B radiation on skin cells of rats was 500mJ/cm^−2^ with an exposure time 12 min per day (daily) for 1 month. The formulations dose 2.0mg cm^−2^ were applied daily 30 min before UV-B irradiation. The dosing of cream and UV-B irradiation was continued for 30 days and rats were sacrificed afterwards. We determined that in our conditions, increased level of end products of lipid peroxidation in skin samples of rats irradiated with UV-B radiation were observed. Table 4 summarizes the increased level of MDA in skin tissue and suggests the increased lipid peroxidation leading to skin tissue damage and failure of defense strategy to prevent formation of ROS in presence of UV-B radiation. In the present work, it was observed that pretreatment of Calendula essential oil based cream formulations (F4 and F5) in the dose of 2mg cm^−2^/day topically decreased the level of MDA.

After UV-B irradiation, SOD level was lowered significantly (p<0.01) when compared with control, but in the case of Gr III and Gr IV, both the formulations significantly (p<0.01) increased the SOD level even more than the control group. There was no significant (p<0.01) difference between Gr I and Gr III SOD level. The F5 formulation was observed to be more significant in increasing the level of SOD. The level of CAT in UV-B treated group animals was found to be lower in comparison to the control group. The F4 and F5 formulation significantly (p<0.01) increased the CAT level but the tendency of F5 to increase the CATe level was more prominent. Also it was observed that the GSH level was significantly (p<0.01) decreased in UV-B induced oxidative stress but the level of GSH was significantly (p<0.05) increased after treatment with F4 and F4 cream. The F5 formulation increased the level of GSH more in comparison to F4 formulation. It was found that UV-B irradiation caused significant (p<0.01) reduction in ASC level when compared with control.

However, both the formulations (F4 and F5) increased the ASC level (Table 5). Decreased TP level revealed susceptibility to UV-B induced oxidation of protein content of skin tissue. Total protein content for UV-B treated group was observed to be significantly (p<0.01) lower as compared to the control group but the F4 and F5 formulations changed this condition (Table 5). There was no significant difference between TP value obtained for Gr I and Gr IV.

Topical application or oral administration of antioxidants has been recently suggested as preventive therapy for skin photoaging and UV-B induced cancer. The level of biochemical parameters as MDA, SOD, CAT, ASC and TP level constitute the defense team against ROS generated in oxidative stress [15]. Formation of ROS and subsequent lipid peroxidation is considered to be mechanism UV radiation induced photodamage. Recent studies have showed that the phytoconstituent, especially flavonoids and terpenoids found in Calendula herb, may be helpful to maintain the skin biological integrity [7, 9].

Studies conducted on essential oil mixtures of thyme or clove leaf with cinnamon leaf, rose, or parsley seed toward skin lipid, squalene oxidized by UV irradiation showed inhibitory activities toward malonaldehyde (MDA) formation [16]. The conventional spectrophotometric methodology for MDA determination requires addition of TBA to the sample and heating at 90°C to form the MDA–(TBA)_2_ complex. Meanwhile, unsaturated fatty acids of biological sample also react with TBA to form colored substance, which also absorb at or near 535 nm. Thus, at the same wavelength, a higher amount of MDA is expected. MDA determination through formation of TBA complexes by the HPLC method also includes heat process and the same problem occurs there. However, sensitivity and specificity is more in the case of HPLC method. In the present study, MDA was estimated as lipid peroxidation marker by conventional spectrophotometric method. In our research, calendula oil based formulations (F4 and F5) when applied on rat skin prior to 30 min of UV-B exposure, caused a significant reduction in MDA level. It may be possible that Calendula based creams exhibit their effect due to the antioxidant effect. SOD, which is one of the cellular antioxidant enzymes, may play a key role as a defensive mechanism against oxidative damage because SOD catalyzes dismutation of O_2_^−^ to O_2_ and H_2_O_2_. Recent studies on biochemistry of the skin tissue have shown marked decreases in SOD activity after exposure to UV-B radiation leaving the cell susceptible to oxidative damage [17]. Increased levels of SOD in formulation treated groups (Gr III and Gr IV) indicated that F4 and F5 formulation may scavenge the ROS generated from UV-B radiation. Recently researchers have suggested that hydrogen peroxide is produced in the body organs naturally as well as by UV-B irradiation and catalase breaks it down [18]. If there is a dip in catalase levels, hydrogen peroxide cannot be broken down. Hydrogen peroxide is a harmful by-product of many normal metabolic processes. To this end, catalase is frequently used by cells to rapidly catalyze the decomposition of hydrogen peroxide into less reactive gaseous oxygen and water molecules [19]. In the present study, after UV-B irradiation, the level of catalase decreased but treatment of both the formulations reversed the condition and increased levels of catalase were observed. Other nonenzymatic antioxidant parameter Glutathione exists in reduced (GSH) and oxidized (GSSG) states. In the reduced state, the thiol group of cysteine is able to donate a reducing equivalent (H^+^+e^−^) to other unstable molecules, such as reactive oxygen species. GSH is considered to be a major endogenous antioxidant produced by the cells which participates directly in the neutralization of free radicals and reactive oxygen compounds, as well as maintains the exogenous antioxidants such as vitamins C and E in their reduced (active) forms [20]. Decreased glutathione level indicates impaired antioxidant enzyme system of the skin cell [21, 22]. In the present investigation, decreased levels of GSH as obtained for Gr II, the formulation F4 and F5 treatment exhibited increased GSH levels. In earlier studies, it was observed that enhanced levels of ascorbic acid could fight against skin ageing [23]. It means ascorbic acid levels of the skin can be severely depleted after UV irradiation. Considering this fact, the ascorbic acid (ASC) level in the present study was estimated. There was no significant difference in ASC level between group IV and group I. It may be due to the property of Calendula essential oil based cream that increases the ascorbic acid level. It is clear from these facts that increased ROS generation can overwhelm antioxidant defense mechanisms, resulting in oxidative stress and oxidative photo damage of proteins and other macromolecules in the skin.

Oxygen radicals generated from UV-B irradiation may cause modifications of the amino acids of proteins that frequently result in functional changes of structural or enzymatic proteins. It indicated that Calendula oil present in cream scavenges the free radicals by showing the increased level of total protein content as compared to UV-B induced oxidative stress. Recent studies on green tea polyphenol (GTP) revealed that GTP treatment inhibits the UV-B induced protein oxidation *in vitro* in human skin fibroblast cells, which supports *in vivo* observations [24].

The realm of possibilities in photo protection may include the development of sunscreens which remain at the surface of the skin for a longer time and may incorporate antioxidants that can neutralize ROS. Herbal compounds such as phenolic acids, flavonoids and polyphenols are very useful in eliminating the adverse effects of UV radiation on the skin. Antioxidants such as reduced glutathione (GSH) and enzymes such as superoxide dismutase (SOD), catalase (CAT), glutathione peroxidase (GPx) are known to attenuate the generation of ROS by removing potential oxidants or by transforming reactive oxygen species and reactive nitrogen species into stable compounds [25]. Hydrophilic cream prepared from Calendula extract proved its significant antioxidant activity and suitable chemical and microbial stability [26]. Findings of this research suggested that cream formulations based on Calendula essential oil have significant potential to protect and maintain the skin biochemical parameter in UV-B induced oxidative stress conditions. The findings also suggested that such formulations may prevent the oxidative decomposition which is responsible for the lipid peroxidation as well as formation of toxic components. The concentration of 1,8-cineole and α-pinene as major terpenoid content found in Calendula essential oil were 8.12 and 22.535 w/w, respectively, which may account for possible mechanism of balanced level of lipid peroxidation and antioxidant enzymes in UV-B induced oxidative damage in skin of albino rats.

In conclusion, the topical application of cream formulations containing 4% (F4) or 5% (F5) of Calendula essential oil before UV-B irradiation can significantly protect the skin from ROS generated from harmful radiation. Lowering in lipid peroxidation marker enzymes indicated the UV-B protective nature of Calendula essential oil based cream. The parameters included in the present investigation cannot reflect the complete damage induced by UV-B radiation. In the future, it will be necessary to conduct studies aiming at the evaluation of systemic effects of topically applied Calendula essential oil based cream will be required.

## Experimental

### Chemicals

The chemicals as glacial acetic acid, Ethylenediaminetetraacetic acid, sodium cyanide, nitroblue tetrazolium, thiobarbituric acid, thiourea, folins ciocalteus, hydrogen peroxide, sodium dodecylsulphate etc. were purchased from Central Drug House (P) Ltd. and Merck India Ltd. 1,8-Cineole and α-pinene as marker compound were procured from Sigma Aldrich Chemie (Steinheim, Germany). Methanol, water and tetrahydrofuran were of HPLC grade and purchased from Merk Chemicals Co. All other reagents were of the highest grade commercially available.

### Procurement of Calendula flowers

The flowers of *Calendula officinalis* for the proposed study were collected from the botanical garden of IFTM; District Moradabad, Uttar Pradesh India in January 2010. The specimens were authenticated and voucher specimen of herbarium (ref. no. IFTM/Pharmacog/Auth/10/2) is preserved in herbarium section of Pharmacy deptt, IFTM, Moradabad.

### Isolation of Calendula oil

The separated petals of flowers were washed thoroughly and then packed in distillation flask of Clavenger’s apparatus with sufficient quantity of water with few porcelain chips to avoid bumping during distillation. The Calendula oil was collected from graduated receiver and purified by anhydrous sodium sulphate for removing water traces.

### HPLC fingerprinting of Calendula essential oil

Fingerprint profile study of Calendula essential oil was performed using an isocratic HPLC system (Shimazdu co.) equipped with a UV detector and the effluent was monitored at 292nm. DS-200 software was used for instrument control, data collection and data processing. The column used was C-18 column (Luna Phenomenex) (250×4mm, 5μm particle size). The mobile phase was combination of Methanol: H_2_O: Tetrahydrofuran(THF) (50:46:4) with a flow rate 1 mL/min. Injection volume for all samples and standard solutions was 20 μL. Standard stock solution of 1,8-cineole (1 mg/mL) and α-pinene (1 mg/mL) were prepared in methanol because 1,8-cineole and α-pinene were insoluble in mobile phase. Dilutions of standard stock solution were done to obtain the concentration in range of 10μg/mL to 100μg/mL and then linear equation was established. Ten milligrams of essential oil was added to a 250 mL volumetric flask and diluted to 100 mL with Methanol : H_2_O : Tetrahydrofuran (THF) (50:46:4). The RTfor 1,8-cineole and α-pinene in this system were 6.8min and 7.32min respectively. The validation of this method was done on parameters including linearity, LOD, LOQ, accuracy and precision (interday and intraday).

### Formulation

An optimized stable cream base was prepared as per method Mishra et al [27]. The components of optimized base formula constituted Stearyl alcohol, Beeswax, Sorbitan monooleate, Sorbitol solution 70%USP, Polysorbate 80. The Calendula oil 4% and 5% (v/v) were incorporated in the previously prepared stable cream base under constant homogenization and code assigned F4 and F5. The formulations were stored in closed amber colored glass bottle.

The chemicals employed in formulations may interfere with the extracted Calendula oil. Therefore, the proposed analytical method was used to assess the recovery of extracted component with special reference to 1,8-cineole and α-pinene from the two formulations. This may be helpful in quality control aspects of formulations.

### Animals

The study was conducted after obtaining institutional animal ethical committee (IAEC) clearance. Albino rats (Wistar strain) were employed in the present study. The animals were maintained at controlled temperature under alternating light and dark conditions, relative humidity (60±5%) and housed in polypropylene cages. Standard food pellets *ad libitum* and drinking water were provided.

### Experimental Groups

To evaluate the effect of Calendula essential oil based cream on lipid peroxidation and antioxidants as well as enzyme level of skin of albino rats against UV-B radiation, the four groups each of six albino rats each were prepared and assigned Gr1, Gr2, Gr3 and Gr4 (Table 6). The back of the animals in each group were shaved 2 days prior to start of the experiment.

### Exposure to UV-B radiation

Two ultraviolet lamps (Toshiba Company) were used to induce photo damage on skin surface of rats. The emission peak was 298nm and the spectral output was in the range of 290–310nm. The dose for radiation exposure (mJ/cm^−2^) was calculated from irradiance (mW cm^−2^) X time of exposure (min). Time of exposure was calculated as per OECD guidelines.

Thirty minutes prior to irradiation, the dose of 2.5mg cm^−2^ cream formulations were applied to the back skin of Gr3 and Gr4 of animals. In the present study, Gr3 and Gr4 animals were irradiated with radiation exposure dose 500mJ/cm^−2^ with an exposure time of 12 min per day (daily) for 1 month. The distance from the lamp and the backs of mice was 20 cm.

After one month of study, rats were sacrificed. The shaved dorsal skin samples were carefully dissected free. Tissue specimens from back of the skin for all the groups were thoroughly rinsed using physiological saline, diluted ten times with distilled water and stored at −70°C. One gram of skin tissue sample was weighed from each group and frozen tissue samples were minced on glass plate over ice bags and then subjected to homogenization. The tissue homogenate was centrifuged (Microcentrifuge-Remi Co.) at 8000rpm for 10 min and supernatant liquid was stored at −80°C in deep freezer until the start of the study of biochemical parameters.

### Biochemical Estimation

#### Estimation of Malondialdehyde (MDA) content

MDA, a terminal product of lipid peroxidation, was measured to estimate the extent of lipid peroxidation. The concentration of MDA in skin was determined by using thiobarbituric acid (TBA) method with little modification [28, 29]. For this, to 0.2 mL of homogenate, 0.2 mL of 8.1% sodium dodecyl sulphate (SDS) and 1.5 mL of 20% acetic acid buffer (pH 3.5) were added. The mixture was centrifuged at 5000rpm for 5 min. The supernatant removed from centrifuge tube was mixed with 1.5 mL of 1% TBA and 1 ml of double distilled water and finally incubated at 90°C for 1 hr and cooled subsequently. For the control solution, in spite of TBA, 1.5 mL double distilled water was used. The absorbance of solution was measured at 532nm. The values of MDA reactive material were expressed in terms of MDA (nmol/mg protein).

#### Estimation of superoxide dismutase (SOD)

The SOD activity in the skin tissue was estimated by method described by Marklund et al. with minor modifications as described by Nandi et al. [30, 31]. The test was performed by monitoring oxidation rate of pyrogallol at 420nm in a reaction medium having 4.5 ml of 100nM Tris–HCl buffer (pH 8.2) and 4.2 mL of doubled distilled water and 300 μL of tissue homogenate. All together were incubated at 25°C for 10 min, immediately adding 0.3 mL of 3mM pyrogallol self oxidation in 10mM HCl or 0.3 mL of 10 mM HCl in case of control at 25°C. Ideally, one unit activity of superoxide dismutase is defined as the amount of enzyme that inhibits 50% self oxidation of pyrogallol in assay conditions. The results were calculated as unit per minute per milligram of protein (IU/mg pr. min).

#### Estimation of catalase (CAT)

The catalase estimation was performed by the method described by Sinha [32]. This used 100 μL of diluted homogenate and 1.0 mL of phosphate buffer along with 0.4 mL of distilled water to which 0.5 mL of H_2_O_2_ solution was added to initiate the reaction, while the H_2_O_2_ solution was left out in control test tubes. After incubating for 1 min at 37°C, 2 mL of potassium dichromate acetic acid was added to stop the reaction. The samples were kept in boiling water bath for 15 min, finally cooled and finally the absorbance measured at 570nm against control. The activity of catalase was calculated as μmol per minute per milligram of protein (μmol/mg pr.min).

#### Estimation of glutathione (GSH)

Reduced glutathione was measured by its reaction with 5,5′-dithiobis(2-nitrobenzoic acid) (DTNB) to give a yellow colored 5-thio-2-nitrobenzoic acid (TNB) compound that absorbs at 412nm (Ellman’s method) [33]. The produced disulfide is reduced by NADPH in the presence of glutathione reductase. 1 mL of tissue homogenate for each sample was taken in corresponding glass tubes. 3 mL of DTNB solution (0.01% in phosphate buffer 0.1M, pH 8) and 750 mL of 0.3 mM NADPH solution (143 mM sodium phosphate and 6.3 mM Na_4_-EDTA; final pH 7.5) were incorporated into each test tube and then were diluted with 4 mL of 0.1M phosphate buffer, pH 8. The absorbance of each solution was determined at 412 nm against a blank which already has 5 mL of phosphate buffer and 3 mL of DTNB and 750 mL NADPH solution. The rate of formation of TNB was measured at 412 nm on a spectrophotometer every 10 s for 50 s, and GSH was quantified by a standard curve taking into account the dilution factor. Value was expressed as nmole/min/mg protein.

#### Estimation of Ascorbic acid (ASC)

Ascorbic acid content was determined by method described by Menon et al. [34]. To the 4 mL of homogenate solution, 1 mL of 2,4-dinitrophenyl hydrazine and a drop of thiourea were added to activate the reaction. For blank solution, 4 mL of 6% tricarboxylic acid (TCA) and in the case of standard, 4 mL of ascorbic acid solution (10 μg/μL) was used. The test tubes were kept in boiling water bath for 15 min and then cooled. Afterwards, 5 mL of sulphuric acid was added to all the samples and allowed to stand for 15 min. The optical density was measured for all the colored samples at 540 nm. The results were expressed in mg of ascorbic level/100 mL.

#### Estimation of Total protein content (TP)

The amount of protein present in all samples was determined by method described by **Lowry** et al. [35]. Different dilutions of bovine albumin (BA) were prepared by mixing stock BA solution (1 mg/mL) and water in the test tubes and finally volume was adjusted 5 mL. From this, 0.2 mL of protein sample was added to different test tubes along with 2 mL of alkaline copper sulphate solution (analytical reagent). The resulting solutions were mixed well and subjected for incubation at 37°C for 10 min. Afterward, 0.2 mL of Folin Ciocalteau solution was added to each test tube and incubated at 37°C for 30 min. Finally optical density was measured at 660nm. Calibration curve was plotted and the concentration of total protein content was estimated by this.

## Figures and Tables

**Tab. 1:** Concentration of 1,8-cineole and α-pinene in Calendula essential oil by HPLC method

Compound	Concentration (%w/w)
1,8-cineole	8.12±0.7
α-pinene	22.53±0.2

**Tab. 2:** HPLC method validation parameters for quantization of 1,8-cineole and α-pinene in *Calendula officinalis* L. essential oil

Parameters	Result	
	
	1,8-cineole	α-pinene
Concentration range (μg/ml)	10–100	10–100
Linearity (r^2^)	0.9989	0.9957
LOD and LOQ	1.8 and 5.5	8.5 and 25.5
Intra-day precission (50 and 60) (n=3, %RSD)	1.2 and 0.8	1.5 and 1.6
Intra-day precission (50 and 60) (n=3, %RSD)	1.4 and 1.2	1.3 and 1.9

**Tab. 3:** Recovery studies of 1,8-cineole and α-pinene from formulations

	F4		F5	
	
	1,8-cineole	α-pinene	1,8-cineole	α-pinene
Mean Recovery (%)±SEM(n=3)	97.5±0.23	97.2±0.62	96.2±0.44	95.8±0.84

**Tab. 4:** Effect of UV-B radiation and the formulations on MDA, SOD and CAT levels in rat skin

Groups	Treatments	MDA content (nmoles/mg±SEM)	SOD (IU/mg±SEM)	CAT (μmole/min/mg±SEM)
Gr I	Control	1.13±0.288^b^	6.32±0.54^b^	45.12±0.24^b^
Gr II	UV-B Irradiated	1.85±0.421^a^	1.32±0.41^a^	20.15±0.45^a^
Gr III	UV-B + F4 treated	1.26±0.211^a^,^b^	6.52±0.52^b^	35.62±0.15^a^,^b^
Gr IV	UV-B + F5 treated	1.18±0.144^a^,^b^	7.11±0.39^a^,^b^	39.99±0.25^a^,^b^

a Significantly different control group I(p<0.01);

b Significantlly different from group II(p<0.05).

The values are presented as Mean±SEM; each group consisted of 6 albino rats.

**Tab. 5:** Effect of UV-B radiation and the formulations on GSH, ASC and TP levels in rat skin

Groups	Treatments	GSH level (nmoles/min/mg)	ASC (mg/100mL±SEM)	TP (μg/mL ±SEM)
Gr I	Control	35.43±0.28^b^	8.11±0.57^b^	626±0. 443^b^
Gr II	UV-B Irradiated	18.25±0. 21^a^	3.19±0.35^a^	465±0.345^a^
Gr III	UV-B + F4 treated	26.45±0.51^a^,^b^	7. 21±0.15^a^^b^	592±0.412^a^,^b^
Gr IV	UV-B + F5 treated	33.65±0. 44^a^,^b^	7.85±0.10^a^,^b^	630±0.24,^b^

a Significantly different control group I(p<0.01);

b Significantlly different from group II(p<0.05).

The values are presented as Mean±SEM; each group consisted of 6 albino rats.

**Tab. 6:** Animal grouping

Groups (n=6)	Treatment
Gr I	Without UV-B Irradiation and without cream treatment
Gr II	UV-B irradiated
Gr III	UV-B +F4 treated
Gr IV	UV-B +F5 treated
